# Interferon-Inducible CXC Chemokines Directly Contribute to Host Defense against Inhalational Anthrax in a Murine Model of Infection

**DOI:** 10.1371/journal.ppat.1001199

**Published:** 2010-11-18

**Authors:** Matthew A. Crawford, Marie D. Burdick, Ian J. Glomski, Anne E. Boyer, John R. Barr, Borna Mehrad, Robert M. Strieter, Molly A. Hughes

**Affiliations:** 1 Department of Medicine, Division of Infectious Diseases, University of Virginia, Charlottesville, Virginia, United States of America; 2 Department of Medicine, Division of Pulmonary & Critical Care Medicine, University of Virginia, Charlottesville, Virginia, United States of America; 3 Department of Microbiology, University of Virginia, Charlottesville, Virginia, United States of America; 4 Centers for Disease Control and Prevention, Atlanta, Georgia, United States of America; The University of Texas-Houston Medical School, United States of America

## Abstract

Chemokines have been found to exert direct, defensin-like antimicrobial activity in vitro, suggesting that, in addition to orchestrating cellular accumulation and activation, chemokines may contribute directly to the innate host response against infection. No observations have been made, however, demonstrating direct chemokine-mediated promotion of host defense in vivo. Here, we show that the murine interferon-inducible CXC chemokines CXCL9, CXCL10, and CXCL11 each exert direct antimicrobial effects in vitro against *Bacillus anthracis* Sterne strain spores and bacilli including disruptions in spore germination and marked reductions in spore and bacilli viability as assessed using CFU determination and a fluorometric assay of metabolic activity. Similar chemokine-mediated antimicrobial activity was also observed against fully virulent Ames strain spores and encapsulated bacilli. Moreover, antibody-mediated neutralization of these CXC chemokines in vivo was found to significantly increase host susceptibility to pulmonary *B. anthracis* infection in a murine model of inhalational anthrax with disease progression characterized by systemic bacterial dissemination, toxemia, and host death. Neutralization of the shared chemokine receptor CXCR3, responsible for mediating cellular recruitment in response to CXCL9, CXCL10, and CXCL11, was not found to increase host susceptibility to inhalational anthrax. Taken together, our data demonstrate a novel, receptor-independent antimicrobial role for the interferon-inducible CXC chemokines in pulmonary innate immunity in vivo. These data also support an immunomodulatory approach for effectively treating and/or preventing pulmonary *B. anthracis* infection, as well as infections caused by pathogenic and potentially, multi-drug resistant bacteria including other spore-forming organisms.

## Author Summary

Innate immunity is critical to host defense and plays a central role in protecting the lungs from respiratory pathogens. Among the mediators important in the innate host response to pulmonary infection are chemokines, proteins originally described for their ability to regulate immune cell trafficking during an inflammatory response. More recently, chemokines have been found to exert direct antimicrobial activity against a broad range of bacteria and fungi in vitro. While these observations suggest chemokines may contribute to host defense by killing microorganisms at local sites of infection through activities not associated with cellular chemokine receptors, the biological relevance of direct chemokine-mediated antimicrobial activity in vivo has not been established. Here we show that the murine chemokines CXCL9, CXCL10, and CXCL11 exert direct antimicrobial effects against *B. anthracis* in vitro and that neutralization of these CXC chemokines, but not their shared receptor CXCR3, increases host susceptibility to pulmonary *B. anthracis* infection in vivo. These data provide unique insight into the host mediators important in host-pathogen interaction and pathogenesis of disease and support the emerging concept that host chemokines mediate efficient, pleiotropic roles that include receptor-independent promotion of host defense in vivo.

## Introduction

The pulmonary airways represent a major site of interaction between the mammalian host and microbial pathogens. Infection resulting from the exposure of the respiratory tract to a variety of microorganisms is opposed by pulmonary innate immunity, a complex host response that protects against infection by directly mediating initial host defense in the airspace while helping to shape the activation of adaptive immunity [1], [2]. Among the primary components of innate immunity are secreted mediators including chemokines, small proteins produced mainly by epithelial and phagocytic cells in response to pattern-recognition receptor engagement and pro-inflammatory cytokines [3]. Chemokines were originally recognized for their ability to induce directed migration of leukocytes and facilitate controlled cellular accumulation and activation during an inflammatory response through receptor-dependent interactions between chemokines and their specific G-protein-coupled receptor(s) expressed by responsive cells [4].

In addition to their role in cellular recruitment, a number of chemokines have been found to mediate direct antimicrobial effects against a broad range of Gram-positive and Gram-negative bacteria in vitro [5]–[8]. While the mechanistic details of these effects remain undefined, antimicrobial activity is thought to result from interactions between positively-charged regions present at the chemokine' C-terminus and negatively-charged moieties at the microbial cell surface, resulting in cell lysis [8]. Although chemokines have been shown to be central components of the host response to pulmonary infection [9], these molecules have primarily been viewed in the context of receptor/ligand interactions, without consideration for direct ligand-mediated antimicrobial activity. As such, the biological relevance of receptor-independent, chemokine-mediated antimicrobial activity in host defense in vivo remains to be established.

The disease anthrax is caused by the Gram-positive, spore forming bacterium *Bacillus anthracis*. The infectious *B. anthracis* spore consists of distinct, concentric layers that encase the spore's genomic material and provide protection against multiple stresses including high temperature and lytic digestion [10], [11]. Depending on the spore's route of entry, *B. anthracis* causes three distinct types of disease: inhalational, gastrointestinal, and cutaneous anthrax. Inhalational anthrax results as a consequence of spore deposition within the host airspace. Here, spores encounter effectors of host innate immunity and are taken up by phagocytes including macrophages [12] and dendritic cells [13]. It is thought that spore germination, the resumption of metabolic activity and outgrowth as a vegetative cell, begins following phagocytosis at these localized sites of infection [14], [15] and that the vast majority of germinating organisms are killed [16]. During transit by phagocytic cells to the regional lymph nodes, however, a small subset of surviving bacilli are believed to mediate membrane disruptive events allowing escape from phagocytic vesicles and, subsequently, the phagocytic cell [17]. Extracellular bacilli evade host immune responses through the production of two principle, plasmid-encoded virulence factors: a tripartite toxin encoded by pXO1 and responsible for broadly suppressing the host immune response [18], and a poly-D-glutamic acid capsule encoded by pXO2, that protects against phagocytic killing [19]. These and other bacterial factors allow *B. anthracis* to multiply rapidly, resulting in systemic dissemination, toxemia, and death of the infected host [20].

CXCL9, CXCL10, and CXCL11 are homologous, interferon-inducible members of the CXC chemokine family that lack the tripeptide structure/function motif Glu-Leu-Arg (ELR) important in neutrophil chemoattraction [9]. As such, these interferon-inducible ELR^-^ CXC chemokines signal through a common receptor, CXCR3, to facilitate selective recruitment of mononuclear leukocytes, natural killer cells, and plasmacytoid dendritic cells to sites of inflammation [9], [21]. We [22] and others [5], [8] have previously reported the ability of human CXCL9, CXCL10, and CXCL11 to exert direct antimicrobial activity against *B. anthracis*, as well as *Escherichia coli*, *Listeria monocytogenes*, and *Staphylococcus aureus*. Furthermore, we have observed that CXC chemokine induction in the lungs of C57BL/6 mice challenged intranasally with *B. anthracis* Sterne strain spores is associated with significant reductions in spore germination and subsequent disease progression [22]. Based on these observations, we hypothesized that murine CXCL9, CXCL10, and CXCL11 exert direct antimicrobial effects against *B. anthracis* and thereby mediate a receptor-independent contribution to host defense against pulmonary *B. anthracis* infection.

In the present study, we demonstrate that the murine interferon-inducible ELR^-^ CXC chemokines CXCL9, CXCL10, and/or CXCL11 exert direct antimicrobial effects against toxigenic, unencapsulated *B. anthracis* Sterne strain (pXO1^+^ pXO2^−^) as well as toxigenic, capsule-forming Ames strain (pXO1^+^ pXO2^+^) spores and bacilli. Furthermore, we show that neutralization of these CXC ligands, but not their shared cellular receptor CXCR3, in C57BL/6 mice challenged with *B. anthracis* Sterne strain spores significantly increases host susceptibility to inhalational anthrax. These observations support that the CXC chemokines directly contribute to host defense against pulmonary *B. anthracis* infection in vivo, providing unique insight into the effector mechanisms of the innate host response to bacterial infection. These data also support the consideration of antimicrobial chemokines in the development of novel, therapeutic strategies for countering multidrug resistant pathogens.

## Results

### Murine CXCL9, CXCL10, and CXCL11 exert direct antimicrobial effects against *B. anthracis* Sterne strain spores and bacilli

As the induction of the interferon-inducible ELR^-^ CXC chemokines within the lungs of spore-challenged mice is associated with resistance to inhalational anthrax [22], we sought to determine whether murine CXCL9, CXCL10, and CXCL11 exert antimicrobial activity against *B. anthracis* Sterne strain. Included in these studies were two control murine CC family chemokines, CCL2 and CCL5, whose molecular weights and basic isoelectric points are similar to the CXC chemokines examined [5]. Disruptions in spore germination and bacterial cell viability were assessed using colony-forming unit (CFU) determination, performed in the presence or absence of heat treatment to differentiate between heat-resistant spores and heat-sensitive bacilli.

**Figure 1:**
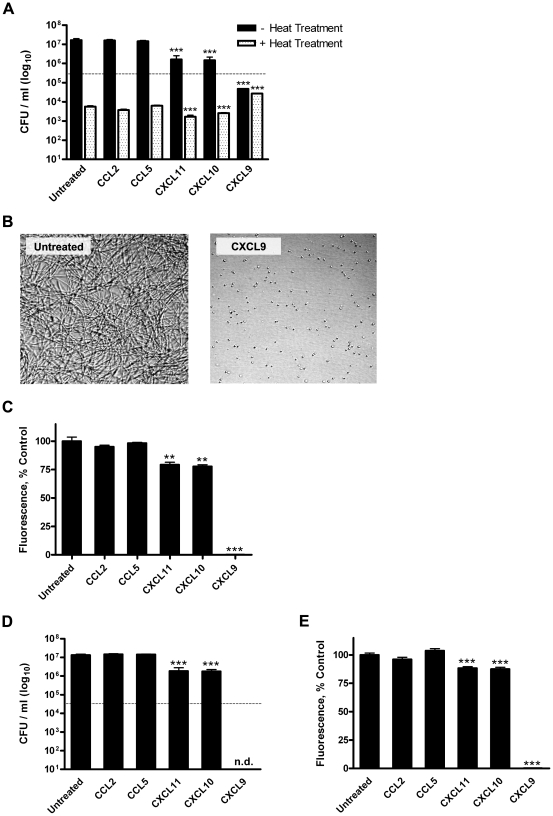
Direct chemokine-mediated antimicrobial effects against *B. anthracis* Sterne strain spores and bacilli. Murine CXCL9, CXCL10, and CXCL11 display direct antimicrobial activity against *B. anthracis* Sterne strain (pXO1^+^ pXO2^−^) organisms. (**A**) Quantification of direct chemokine-mediated disruptions of Sterne strain spore germination, viability, and primary outgrowth. CFU determination was performed 6 h post-treatment in the presence or absence of heat treatment. Data represent mean ± SEM; dotted line indicates the initial inoculum. Similar results were observed in three independent experiments. ***p<0.001 compared to untreated control. (**B**) Microscopic visualization of untreated and CXCL9-treated *B. anthracis* spores 6 h post-treatment. Representative fields from six independent experiments are shown at 200× magnification. (**C**) Metabolic activity as an index of spore germination, viability, and vegetative growth. Alamar Blue reduction was measured 6 h post-treatment and is expressed as percent control; bars represent mean ± SEM for three independent experiments. **p<0.01 ***p<0.001 compared to untreated control. (**D** and **E**) Chemokine-mediated antimicrobial effects against *B. anthracis* Sterne strain bacilli as measured by CFU determination (**D**) and Alamar Blue analysis (**E**). CFU data represent the mean ± SEM. A representative data set is shown from three separate experiments; initial inoculum (dotted line), n.d.  =  none detected. Alamar Blue data are expressed as percent control and represent mean values ± SEM for three independent experiments. ***p<0.001 compared to untreated control.

By 6 h post-treatment, untreated, CCL2- and CCL5-treated spores underwent considerable germination and vegetative outgrowth as evidenced by a loss of heat-resistant CFU (germination) and an increase in heat-sensitive CFU (vegetative growth) as compared to the initial inoculum (**Figure 1A**). Treatment of *B. anthracis* spores with CXCL9 resulted in an approximate 1,000-fold reduction in viable organisms as compared to the untreated control. Retention of spore dormancy was observed to be significantly greater in the presence of CXCL9, yet viable spores represented less than 10% of the initial inoculum; interestingly, this reduction in viable spores did not coincide with an appearance of heat-sensitive, germinated organisms. These effects were concentration dependent (EC_50_  = 5.00±1.10 µg/ml; **Figure S1A,B**) and suggest that CXCL9 inhibits spore germination and disrupts the maintenance of spore viability. In support of this notion, CXCL9-treated spores demonstrated a lack of primary outgrowth as determined by microscopic visualization (**Figure 1B**), and the resumption of metabolism (a hallmark of spore germination) was absent in CXCL9-treated spore samples 6 h post-treatment as measured by an Alamar Blue based assay of metabolism (**Figure 1C**). Treatment of *B. anthracis* spores with CXCL10 or CXCL11 resulted in significantly decreased levels of heat-resistant organisms and an approximate 10-fold reduction in vegetative outgrowth as compared to the untreated control (**Figure 1A**). While possibly exerting a sporicidal effect, neither CXCL10 or CXCL11 was found to block spore germination (**Figure 1A,C**). Chemokine-mediated antimicrobial activity against *B. anthracis* Sterne strain bacilli was also observed, with all three interferon-inducible ELR^-^ CXC chemokines capable of mediating significant decreases in vegetative cell viability as determined by both CFU analysis (**Figure 1D**) and Alamar Blue reduction (**Figure 1E**). CXCL9 demonstrated considerable bactericidal activity, mediating the complete killing of the initial bacilli inoculum in a concentration-dependent manner (EC_50_  = 3.96±0.75 µg/ml; **Figure S1C,D**). Of note, the antimicrobial hierarchy of the murine CXC chemokines presented here (CXCL9 >> CXCL10 ≈ CXCL11) is distinct from the hierarchy previously observed for the human CXC chemokines (CXCL10≥ CXCL9 >> CXCL11) [22].

### Antimicrobial effects of interferon-inducible ELR^-^ CXC chemokines against *B. anthracis* Ames strain spores and encapsulated bacilli

In contrast to *B. anthracis* Sterne strain organisms, vegetative Ames strain bacilli carry the capsule biosynthetic operon encoded by pXO2 and are capable of generating a protective poly-D-glutamic acid capsule. While the increased virulence of encapsulated organisms has primarily been attributed to enhanced bacterial evasion of cell-mediated host responses [19], the capsule may act as a barrier against soluble immune mediators. Therefore, the antimicrobial potential of murine CXCL9 against fully virulent *B. anthracis* Ames strain organisms was examined using CFU determination. Also, as the ability of human ELR^-^ interferon-inducible CXC chemokines to mediate antimicrobial effects against *B. anthracis* Ames strain is unknown, we sought to determine the capacity of human CXCL10 to directly target fully virulent spores and encapsulated bacilli relevant to human disease; human CXCL10 has previously been shown to exert antimicrobial effects against *B. anthracis* Sterne strain organisms similar to those reported here for murine CXCL9 [22].

**Figure 2:**
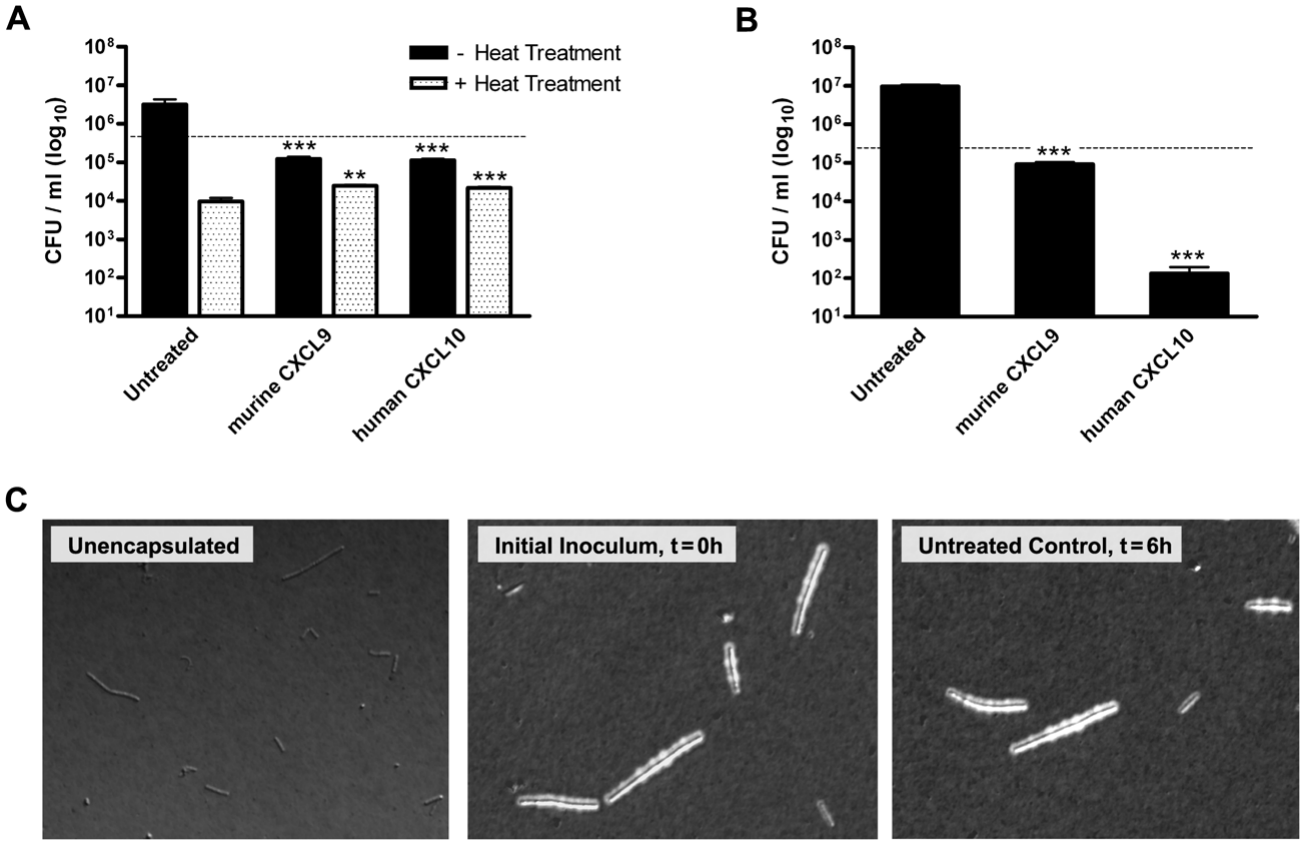
Chemokine-mediated antimicrobial activity against *B. anthracis* Ames strain spores and encapsulated bacilli. Murine CXCL9 and human CXCL10 display direct antimicrobial activity against *B. anthracis* Ames strain (pXO1^+^ pXO2^+^) organisms. (**A**) Chemokine-mediated effects on Ames strain spore germination, viability, and primary outgrowth. CFU determination was performed 6 h post-treatment in the presence or absence of heat treatment. Data represent mean ± SEM; dotted line indicates initial inoculum. A representative data set is shown from three independent experiments. **p<0.01 ***p<0.001 compared to untreated control. (**B**) Direct antimicrobial effects of murine CXCL9 and human CXCL10 against encapsulated Ames strain bacilli. Bacterial cell viability was measured using CFU determination performed 6 h post-treatment. Data represent mean values ± SEM; initial inoculum (dotted line). Similar results were observed in three independent experiments. ***p<0.001 compared to untreated control. (**C**) Microscopic visualization of encapsulated Ames strain bacilli in India ink preparations. Capsules appear as defined clear zones around the bacterial cells. Representative fields from two independent experiments are shown at 200× magnification.

Treatment of Ames strain spores with murine CXCL9 or human CXCL10 was found to result in significantly reduced levels of spore germination and primary outgrowth as compared to the untreated control (**Figure 2A**), supporting a lack of a role for pXO2-encoded components in spore susceptibility to these chemokines. We next examined the ability of murine CXCL9 and human CXCL10 to exert a direct bactericidal effect against toxigenic, encapsulated bacilli. Treatment of encapsulated Ames strain bacilli with murine CXCL9 resulted in an approximate 100-fold reduction in bacterial viability as compared to the untreated control 6 h post-treatment (**Figure 2B**). Similarly, human CXCL10 was found to display antimicrobial activity against encapsulated bacteria, mediating a five-log reduction in viable vegetative cells. Ames strain bacilli were visualized in India ink preparations, confirming that the initial bacilli inoculum consisted of encapsulated bacterial cells, and that the capsule was not lost under experimental conditions (**Figure 2C**). That murine CXCL9 and human CXCL10 exert direct antimicrobial effects against Ames strain spores and encapsulated bacilli, and that these effects are similar to those observed for Sterne strain organisms indicate *B. anthracis* Sterne strain is an appropriate model organism for studying chemokine-mediated antimicrobial activity against this pathogen.

### The interferon-inducible ELR^-^ CXC chemokines directly contribute to host defense against pulmonary *B. anthracis* infection

In order to determine the biological relevance of direct chemokine-mediated antimicrobial activity during pulmonary *B. anthracis* infection, we used a murine model of inhalational anthrax in which endogenous CXCL9, CXCL10, and/or CXCL11, or their shared cellular receptor CXCR3 were selectively neutralized. Antibody-mediated neutralization was performed in C57BL/6 mice (relatively resistant to inhalational infection by *B. anthracis* Sterne strain) and was achieved through intraperitoneal (i.p.) administration of anti-sera raised against individual interferon-inducible ELR^-^ CXC chemokines or the NH_2_ terminus of CXCR3 [23], [24]. These antibodies were previously shown to be specific without cross-reactivity to a panel of cytokines and other chemokine ligands [23].

**Figure 3:**
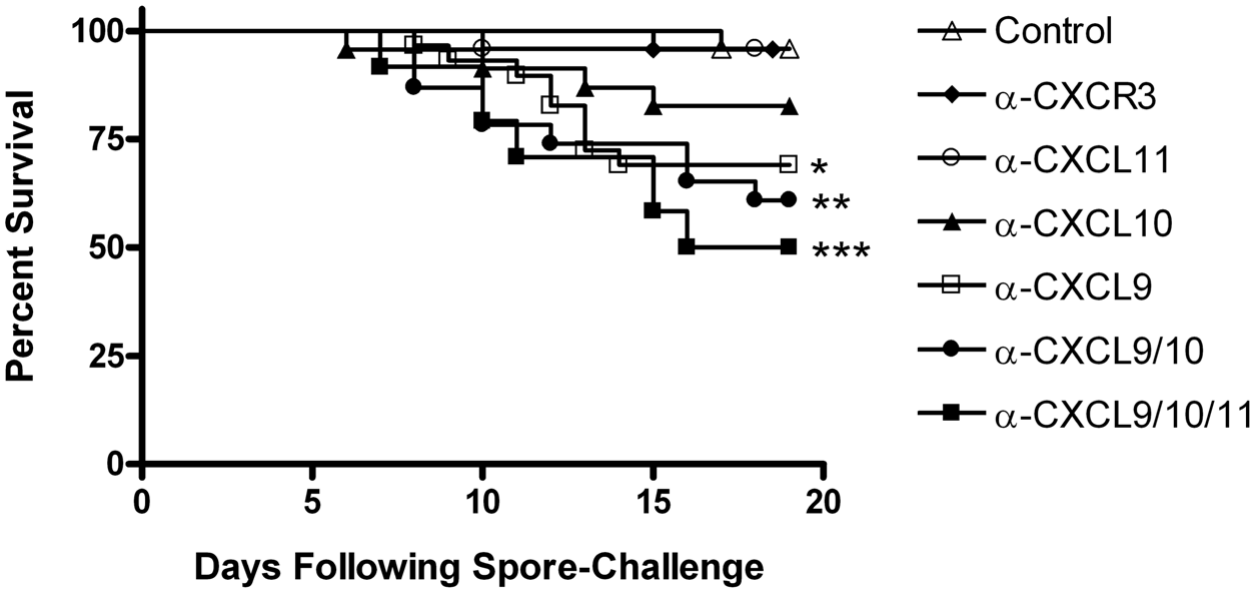
Host susceptibility to pulmonary *B. anthracis* infection. Neutralization of the interferon-inducible ELR^-^ CXC chemokines, but not their shared cellular receptor, significantly increases host susceptibility to inhalational anthrax. Mortality was measured among C57BL/6 mice challenged intranasally with *B. anthracis* Sterne strain spores and receiving control serum or neutralizing serum against the indicated CXC chemokine ligand(s) or their receptor CXCR3. Kaplan-Meier survival curve represents combined mortality from ≥ two independent challenges of 8–12 animals per group, 24–32 animals total per group. Similar results were observed in each independent experiment. *p<0.05 **p<0.01 ***p<0.001 as compared to spore-challenged animals receiving control serum.

Neutralization of endogenous CXCL9 in *B. anthracis* spore-challenged animals was found to significantly increase host susceptibility to pulmonary infection (*p* = 0.012) resulting in approximately 30% mortality as compared to spore-challenged animals receiving control serum, <5% mortality (**Figure 3**); administration of CXCL9 neutralizing serum in the absence of infection was not found to cause death, with 12/12 mice surviving beyond 20 days. Neutralization of CXCL10 in spore-challenged animals resulted in decreased host survival (20% mortality) that approached statistical significance (*p* = 0.064) when compared to infected animals receiving control serum; similar mortality was observed in *CXCL10* ^-/-^ mice following spore challenge (data not shown). CXCL11 neutralization was not found to increase host susceptibility to inhalational anthrax. Combinatorial neutralization of CXCL9 together with CXCL10 or CXCL10/CXCL11 during pulmonary *B. anthracis* infection significantly increased host susceptibility to anthrax, with neutralization of all three CXC chemokines resulting in 50% mortality (*p* = 0.0003).

Importantly, antibody-mediated neutralization of CXCR3 (**Figure S2**) did not result in increased susceptibility to pulmonary *B. anthracis* infection (**Figure 3**), and survival among spore-challenged *CXCR3* ^-/-^ and wild-type animals was the same (data not shown). These data suggest that direct ligand-mediated effects not associated with CXCR3 contribute to limiting disease progression in this model of pulmonary infection. Indeed, the post-challenge induction of endogenous CXCL9, CXCL10, and CXCL11 previously associated with resistance to inhalational *B. anthracis* infection [22] was maintained in animals receiving CXCR3 neutralizing serum (**Figure S3**). Additionally, host inflammatory cell populations in the lungs of spore-challenged animals receiving CXCL9/CXCL10/CXCL11 or CXCR3 neutralizing sera were strikingly similar following challenge (**Figure S4**); the absence of significant differences in host cell populations indicate that CXCR3-dependent, cell-mediated effects are not responsible for the distinct differences in disease progression between these groups. Taken together, the above data demonstrate a novel antimicrobial role for the interferon-inducible ELR^-^ CXC chemokines during pulmonary *B. anthracis* infection that is independent of CXCR3-mediated cellular recruitment to sites of infection.

### Interferon-inducible ELR^-^ CXC chemokine neutralization is associated with *B. anthracis* dissemination and toxemia

To gain insight into disease progression associated with the neutralization of CXCL9, CXCL10, and CXCL11, and to confirm that host death resulted as a consequence of *B. anthracis* infection, we investigated two salient features of anthrax: bacterial dissemination and toxemia. The ability of *B. anthracis* to disseminate from initial sites of infection was examined by measuring *B. anthracis* CFU in the lungs, kidneys, spleen, and liver from moribund mice receiving CXCL9/CXCL10/CXCL11 neutralizing sera, as compared to those measured from paired, spore-challenged animals receiving control serum.

**Figure 4:**
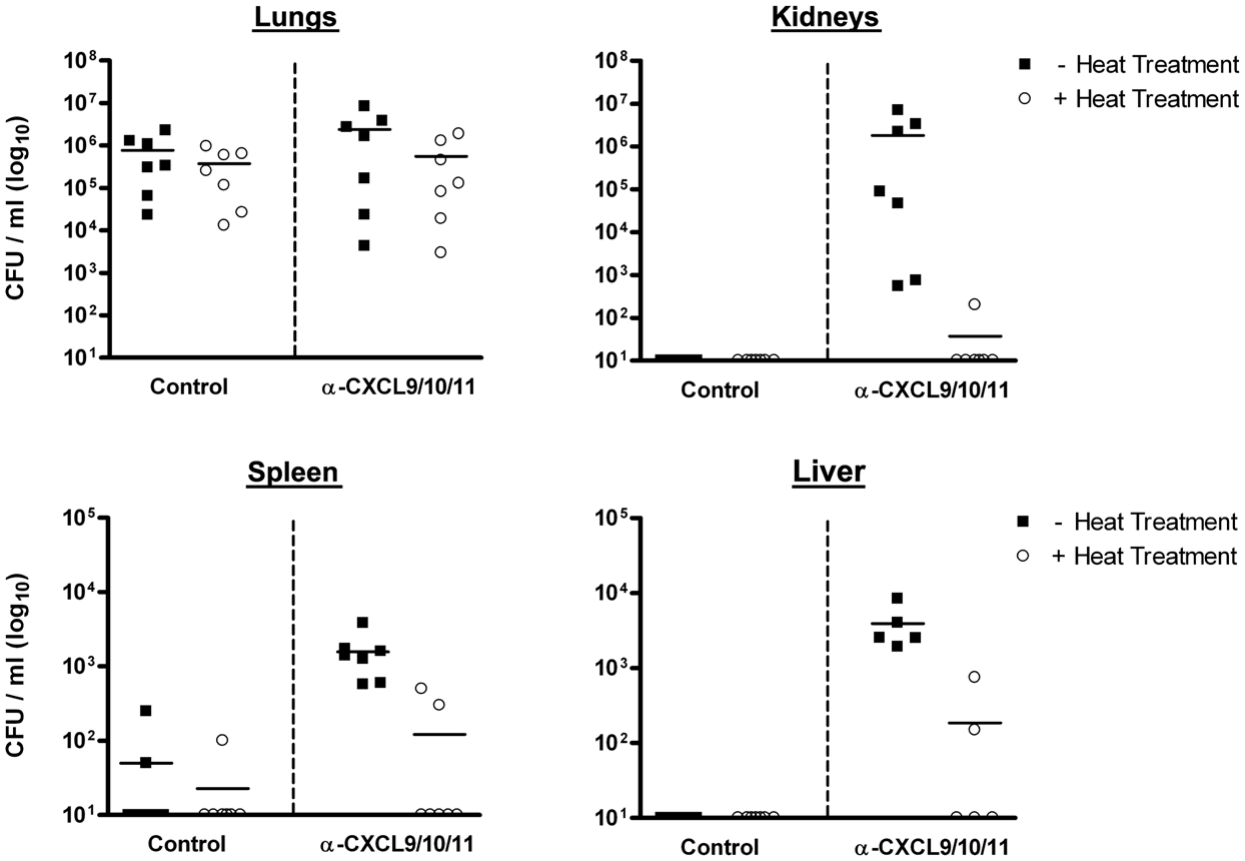
Dissemination of *B. anthracis* from initial sites of infection in the airways. Increased host susceptibility to inhalational anthrax upon neutralization of CXCL9, CXCL10, and CXCL11 is characterized by widespread bacterial dissemination following spore challenge as detected and measured using CFU determination. *B. anthracis* titers in the lungs, kidneys, spleen, and liver were measured from moribund animals receiving CXCL9/CXCL10/CXCL11 neutralizing sera and from paired, spore-challenged animals receiving control serum (5–7 animals per group). CFU values from individual mice are shown for the indicated tissue ± heat treatment; solid lines represent the median for each group. Data points along the X-axis represent values below the limit of detection, 50 CFU/ml. For each tissue examined, median chemokine-neutralized CFU values in the absence of heat treatment were significantly greater than those determined for control mice (*p*<0.01), except lung (*p* = 0.5).

**Figure 5:**
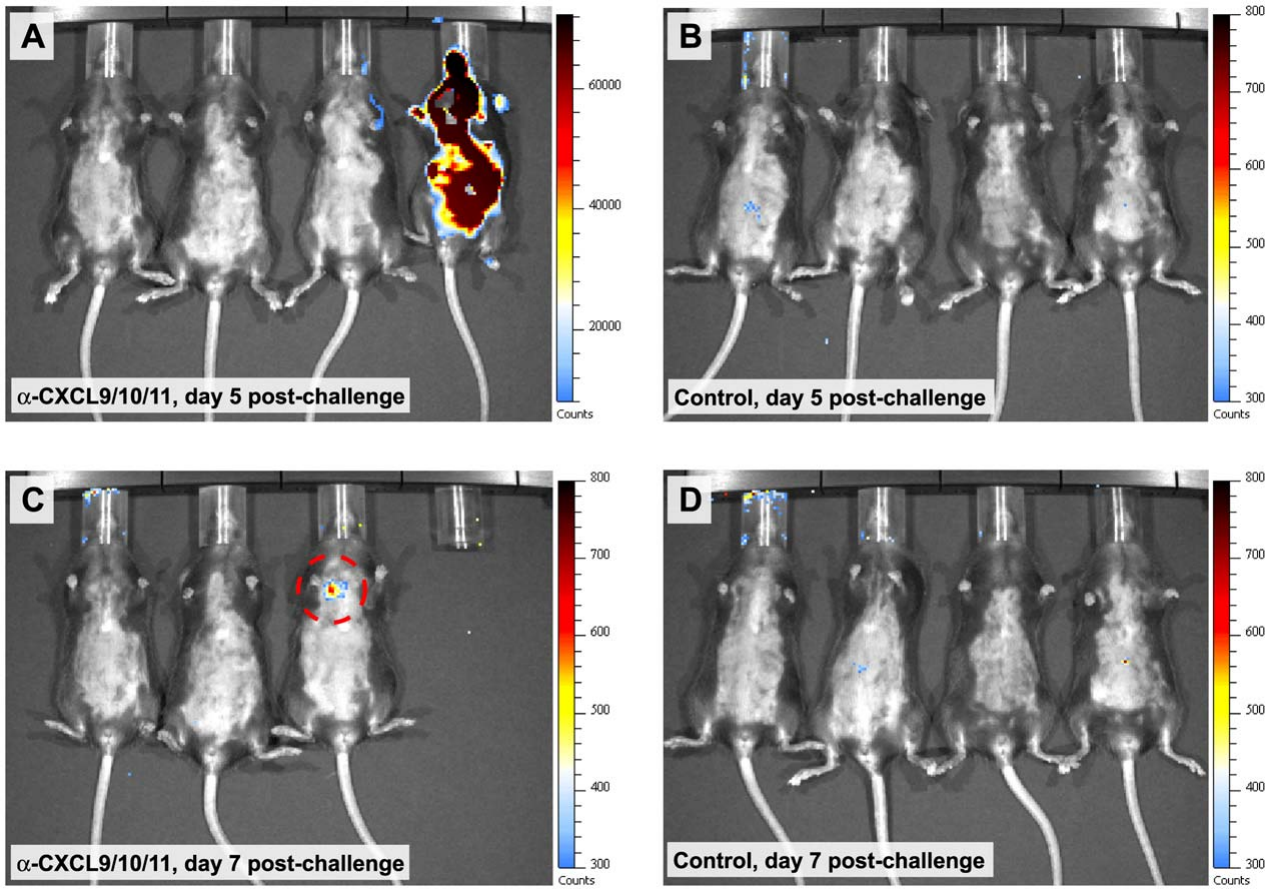
In vivo imaging of *B. anthracis* dissemination in animals receiving CXCL9/CXCL10/CXCL11 neutralizing sera. (**A–D**) Bioluminescence detected and measured in groups of mice challenged intranasally with *B. anthracis* 7702-*lux*. Animals receiving CXCL9/CXCL10/CXCL11 neutralizing sera demonstrated considerable bacterial dissemination as evidenced by the detection of luminescence in multiple organs (panel **A**, animal 4). Systemic dissemination was found to occur after the establishment of localized infection (panel **C**, animal 3; dashed circle). Bioluminescence was not observed in paired, spore-challenged animals receiving control serum (panels **B** and **D**). Animals were imaged every other day for 10 d post-challenge; merged luminescent and photographic images are shown. Luminescence is reported as photons per second per square centimeter per steradian (n = 12 animals per group).

Consistent with previously published reports measuring bacterial dissemination during pulmonary *B. anthracis* infection [25], [26], *B. anthracis* CFU in the lungs of spore-challenged animals were approximately equivalent between treatment groups. However, in contrast to control animals, which showed little evidence of extrapulmonary dissemination, tissues harvested from animals receiving CXCL9/CXCL10/CXCL11 neutralizing sera demonstrated widespread bacterial dissemination with considerable CFU detected in the kidneys, spleen, and liver (**Figure 4**). This observed dissemination is consistent with bacterial dissemination previously reported for strains of mice highly susceptible to Sterne strain infection [20]. The ability of the CXC chemokines to participate in limiting disease progression prior to systemic invasion was also observed using in vivo imaging. C57BL/6 mice were challenged with a bioluminescent strain of *B. anthracis* (7702-*lux*) whose vegetative cells are constitutively luminescent and allow visualization of bacterial dissemination [27]. As above, only upon neutralization of CXCL9, CXCL10, and CXCL11 was systemic disease observed as evidenced by detection of luminescence in tissues distant to the host airways (**Figure 5A,B**). Also, extrapulmonary dissemination was observed to occur after the establishment of infection in the chest (**Figure C,D**) consistent with impaired host defense at local sites of infection.

**Figure 6:**
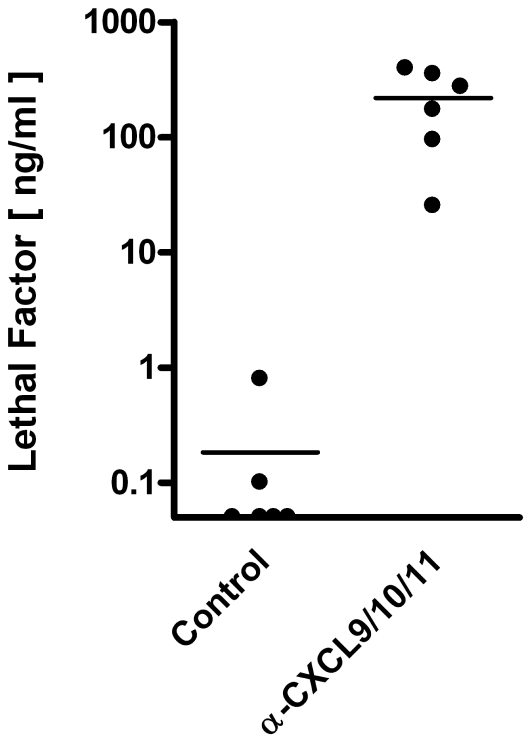
Detection and quantification of toxemia in spore-challenged mice. Quantitative measurement of the *B. anthracis* toxin component LF in sera collected from moribund animals receiving CXCL9/CXCL10/CXCL11 neutralizing sera and paired, spore-challenged animals receiving control serum. Analysis was performed using MALDI-TOF MS with internal standards (see Materials and Methods). Concentrations of active LF measured from individual mice are shown; solid lines represent the median concentration for each group (n = 6 animals per group). LF concentrations measured in sera collected from chemokine-neutralized animals were significantly greater than concentrations determined for control mice (*p* = 0.002). Data points along the X-axis represent values below the limit of detection, 0.005 ng/ml.

Toxemia is characteristic of systemic anthrax and results from the secretion of a tripartite toxin consisting of the receptor binding component protective antigen (PA), and two catalytically active components, the metalloprotease lethal factor (LF) and the adenylate cyclase edema factor (EF) [18]. Several animal studies examining the production of PA and LF during infection have found PA to be detectable in the blood of infected animals only during the terminal stages of disease [28], an observation thought to reflect rapid binding of PA by host cells [29]. Conversely, LF has been shown to accumulate earlier in infection, consistent with delayed internalization (cellular entry of LF depends upon prior PA binding, activation, and heptamerization), providing a good measure of toxemia during disease progression [28]. As an index of toxemia, we used an established mass spectrometry-based method [30] to detect and measure the levels of biologically active LF in serum collected from spore-challenged mice. Whereas infected control animals showed low or undetectable levels of LF, serum collected from spore-challenged animals receiving CXCL9/CXCL10/CXCL11 neutralizing sera was found to contain concentrations of active LF ranging from 25–400 ng/ml (**Figure 6**), levels commensurate with concentrations measured from the sera of nonhuman primates that have succumbed to inhalational anthrax [30]. These data indicate that the interferon-inducible ELR^-^ CXC chemokines help protect against pulmonary *B. anthracis* infection in a murine model of infection, and that disruption of innate, ligand-mediated roles in host defense increases susceptibility to invasive disease and toxemia.

## Discussion

Exposure of the host lungs to potentially pathogenic microorganisms represents a significant immunological challenge for host defense. Initial encounters between inhaled microbes and components of pulmonary innate immunity initiate a dynamic set of interactions that ultimately determines whether disease will occur [31]. The host response to infection is coordinated, in part, through the production of chemokines that allow controlled cellular accumulation and activation during an immune response [32]. Some chemokine ligands display direct antimicrobial activity in vitro [33] raising the possibility of a multifunctional role for chemokines in host defense that includes microbial killing at local sites of host-pathogen interaction. Here, we investigated the ability of the interferon-inducible ELR^-^ CXC chemokines to directly contribute to host defense against pulmonary *B. anthracis* infection. We found that CXCL9, CXCL10, and CXCL11 each exert direct antimicrobial effects against *B. anthracis* in vitro and that neutralization of endogenous CXCL9, individually or together with CXCL10 or CXCL10/CXCL11, but not CXCR3, significantly increases host susceptibility to inhalational anthrax in a murine model of infection. Our data support a novel, CXCR3-independent role for the interferon-inducible ELR^-^ CXC chemokines in the innate host response against pulmonary *B. anthracis* infection that is consistent with direct chemokine-mediated antimicrobial activity at local sites of infection.

Each murine CXC chemokine examined in vitro for antimicrobial activity was found to exert direct antimicrobial effects against *B. anthracis* Sterne strain spores and bacilli. In vitro analysis also demonstrated the ability of murine CXCL9 and human CXCL10 to exert direct antimicrobial effects against fully virulent *B. anthracis* Ames strain spores and encapsulated bacilli. Interestingly, while Ames strain spores were found to be fully susceptible to murine CXCL9, direct killing of encapsulated bacilli by murine CXCL9 was reduced compared to unencapsulated organisms. As CXCL9 contains a relatively extended C-terminal region [5], the relative reduction in antimicrobial activity may result from greater exclusion of CXCL9 by the poly-D-glutamic acid capsule thereby preventing the chemokine from reaching the presumed site(s) of action at the bacterial surface. While this difference may impact the defensive role of CXCL9 during infection with fully virulent *B. anthracis*, the in vitro data presented here demonstrate that chemokine-mediated antimicrobial activity is applicable to both strains of *B. anthracis* examined. Given that the activity of many antimicrobial chemokines and host peptides is disrupted by the presence of serum and/or physiological concentrations of ions including Na^+^, K^+^, and Mg^2+^ [33], [34], it is important to note that the in vitro antimicrobial activity of CXCL9, CXCL10, and CXCL11 against *B. anthracis* was tested in culture medium containing physiologically relevant concentrations of serum proteins and ions. In addition, these ion concentrations are similar to those found in airway surface fluid [35], supporting the potential of the interferon-inducible ELR^-^ CXC chemokines to mediate antimicrobial activity in the host airways.

Previous work by our laboratory has demonstrated that the induction of the interferon-inducible ELR^-^ CXC chemokines within the lungs following *B. anthracis* spore challenge is associated with significant reductions in spore germination and resistance to pulmonary infection [22]. In the present study, we investigated the consequences of selectively neutralizing CXCL9, CXCL10, and/or CXCL11 during pulmonary *B. anthracis* infection, and whether potential ligand-mediated contributions to host defense were independent of interactions with CXCR3. Consistent with its potent antimicrobial activity in vitro and its sustained induction within the lungs following spore challenge in vivo (**Figure S3**; [22]), neutralization of endogenous CXCL9 resulted in significantly increased host susceptibility to inhalational anthrax. While individual neutralization of CXCL10 or CXCL11 was not found to result in significantly increased mortality among spore-challenged animals, combined neutralization of CXCL9 together with CXCL10 or CXCL10/CXCL11 indicated potential additive effects in promoting host defense against pulmonary *B. anthracis* infection, with neutralization of all three CXC ligands resulting in widespread bacterial dissemination, toxemia, and the highest mortality of any spore-challenged group examined in this study. Importantly, CXCR3 neutralization, which disrupts receptor-mediated cellular recruitment in response to these CXC chemokines, was not found to increase host susceptibility to inhalational anthrax. These results demonstrate the ability of the interferon-inducible ELR^-^ CXC chemokines, in particular CXCL9, to contribute directly to host defense through activities not associated with CXCR3. Moreover, these observations support the potential of an efficient, multifunctional role for host chemokines that may represent a more generalized mechanism of the innate host response against infection.

While the data presented here are consistent with direct chemokine-mediated antimicrobial activity in vivo, chemokine ligand concentrations measured from lung homogenates of spore-challenged animals are not as high as those required to achieve antimicrobial effects in vitro. In fact, with few exceptions, most known antimicrobial chemokines and host peptides, including many defensins, exert direct bactericidal effects in vitro at relatively high concentrations; minimal inhibitory concentrations typically range from 0.1–100 µg/ml [36]. Numerous studies, however, have identified roles for antimicrobial host peptides in pulmonary defense against bacterial infection suggesting biologically relevant concentrations do occur during infection [37]. The ability of the interferon-inducible ELR^-^ CXC chemokines to mediate direct antimicrobial activity in vivo is most likely relevant at local sites of host-pathogen interaction. At these inflammatory foci, the elaboration of chemokine production by host cells can be expected to result in substantial chemokine concentrations capable of mediating direct contributions to host defense [1]. This notion is supported by the ability of epithelial [38], [39] and mononuclear cells [5] to produce significant amounts of CXCL9, CXCL10, and/or CXCL11 in response to inflammatory stimuli, with concentrations of CXCL9 and CXCL10 reaching several hundred nanograms per milliliter [39]. Furthermore, tonsil fluid collected from patients with *Streptococcus pyogenes* pharyngitis contains CXCL9 concentrations exceeding those required to kill *S. pyogenes* in vitro, and the inhibition of CXCL9 expression reduces antimicrobial activity against this organism at the surface of inflamed pharyngeal cells [38]. CXCL9 may be of particular importance in promoting host defense against bacterial infection as it is strongly induced in several murine models of pulmonary infection including *Klebsiella pneumoniae* and *Mycobacterium tuberculosis* [9], [40]. Similarly, and of particular relevance to the current study, adults exposed to *B. anthracis* spores (based on positive nasopharyngeal swab cultures) in the U.S. Capitol building during the 2001 anthrax attacks demonstrated elevated levels of several inflammatory mediators including CXCL9 [41].

The ability of CXCL9 to mediate a multifunctional role in host defense is supported by observations that *S. pyogenes* and the opportunistic pathogen *Finegoldia magna* each release specific virulence factors believed to promote immune evasion by disrupting the integrity or availability of the C-terminal region of CXCL9, thereby reducing or abolishing direct antimicrobial activity [38], [42]. Interestingly, while these factors limit CXCL9-mediated antimicrobial activity, the ability of CXCL9 to signal through CXCR3 is largely retained, demonstrating separate and distinct chemokine-mediated functions independently disrupted by pathogens. That other antimicrobial chemokines are similarly targeted [43] further indicates that endogenously produced host chemokines mediate multifunctional roles in host defense that likely represent a more generalized mechanism of the innate host response to infection. Indeed, murine CCL6 and its human homologs were recently found to be highly expressed in the intestinal mucosa and capable of mediating antimicrobial effects against a subset of intestinal bacteria ex vivo [44]. In addition, the antimicrobial chemokine CCL28 has been found to be constitutively expressed and highly concentrated in mucosal secretions [6], and CXCL9 from seminal plasma possesses antimicrobial activity against the urogenital pathogen *Neisseria gonorrhoeae* [45]. These observations are each consistent with direct chemokine-mediated roles in host defense and support the notion of host chemokines as multifunctional effectors of innate immunity.

It remains to be determined at what point in pulmonary *B. anthracis* infection the interferon-inducible ELR^-^ CXC chemokines mediate their contribution(s) to host defense. CXCL9 and CXCL10 are each induced to relatively high levels within the lungs following spore challenge suggesting that antimicrobial activity may act early in infection against the spore form of the organism. Antimicrobial activity against *B. anthracis* spores during infection is consistent with the previously reported association between CXCL9, CXCL10, and CXCL11 induction and decreased spore germination in vivo [22], as well as observations that the reduction of spore burden on resident macrophages is important in preventing intracellular vegetative outgrowth and subsequent disease progression [46]. Spore challenge with toxigenic, unencapsulated *B. anthracis* results in spore germination and the establishment of infection at local sites within the host airways [14], [15]. Infection is initially contained here providing an opportunity for chemokine-mediated antimicrobial activity against vegetative bacilli prior to extrapulmonary dissemination [20]. While the observations reported here are consistent with direct antimicrobial effects similar to those found in vitro, they do not preclude ligand-mediated immunomodulatory activity; CXCL9 has recently been reported to induce gene transcription and chemokine production in peripheral blood mononuclear cells, independent of interactions with CXCR3 [47]. As inhalational anthrax is an acute disease capable of abrogating host immune responses suggests that host chemokines mediate important roles in the innate host response of naïve hosts and help to limit infection early in disease progression.

The continuing emergence of antibiotic resistance [48] and the potential of engineered resistance in the weaponization of biological agents [49] represent serious areas of concern. The ability of host defense peptides to exert direct antimicrobial effects and promote protective immunity has been suggested as a template for the development of novel therapeutic strategies capable of addressing these challenges [50]. Certain chemokines (including CXCL9, CXCL10, and CXCL11) share many structural and functional relationships with host defense peptides, suggesting that these mediators have overlapping roles in host defense and similar therapeutic potential [51]. The ability of type 1 (IFN-α/β) and type 2 (IFN-γ) interferons to strongly induce ELR^-^ CXC chemokine production supports the administration of exogenous interferon as a therapeutic strategy for treating pulmonary *B. anthracis* infection. Indeed, both IFN-α/β and IFN-γ have been found to promote protection against *B. anthracis* challenge *in vitro* [52] and *in vivo* [53]. While neither of these studies examined CXC chemokines, each supports the potential therapeutic application of exogenous chemokine induction in post-exposure prophylaxis or the treatment of anthrax. Furthermore, the exogenous induction of host chemokines capable of activating cellular immunity, promoting immune mediator production, and directly killing pathogens may apply more broadly to the development of innovative therapeutic avenues for the treatment of pathogenic and potentially, multidrug-resistant bacterial infections.

In summary, our findings provide strong evidence for an important CXCR3-independent role for the interferon-inducible ELR^-^ CXC chemokines in the innate host response against pulmonary *B. anthracis* infection, and indicate that CXCL9, in particular, may function as one of the major antimicrobial components of the inflamed host airway. Neutralization of the CXC chemokine ligands, but not their shared cellular receptor, was found to disrupt the host's ability to limit disease progression and contain *B. anthracis* at initial sites of infection, resulting in increased susceptibility to inhalational anthrax characterized by systemic dissemination, toxemia, and death. While further studies are required to define the biologically relevant contributions of the interferon-inducible ELR^-^ CXC chemokines to host defense, the ability of an intact host chemokine response to directly promote the innate host response against inhalational anthrax is consistent with direct antimicrobial activity as observed for these chemokines in vitro. Direct chemokine-mediated antimicrobial activity at the interface of host-pathogen interaction may represent an important mechanism in host defense, and supports the consideration of host chemokines in the development of novel, immunomodulatory therapeutic strategies.

## Materials and Methods

### Ethics statement

Animal studies were carried out in strict accordance with the US Public Health Service Policy on the Humane Care and Use of Laboratory Animals (PHS Assurance #A3245-01), the US Department of Agriculture Animal Welfare Act (USDA Registration #52-R-0011), and the US Government Principles for the Utilization and Care of Vertebrate Animals Used in Testing, Research, and Training. Animal protocols were reviewed and approved by the Institutional Animal Care and Use Committee (IACUC) of the University of Virginia (Protocol #3677).

### Bacterial strains and culture conditions

*B. anthracis* Sterne strain 7702 spores were prepared using a liquid culture method [54] with modification. Briefly, Difco Sporulation Medium [55] was inoculated with *B. anthracis* 7702, and cultures were incubated 4–5 d at 37°C with shaking. After sporulation, cultures were washed in cold, sterile dH_2_O and heat treated at 65°C to kill any remaining vegetative cells. Spores were purified over a Percoll gradient (GE Healthcare Biosciences, Piscataway, NJ, USA) washed, and enumerated. *B. anthracis* bacilli were prepared in brain heart infusion (BHI) broth (Becton, Dickinson and Company, Franklin Lakes, NJ, USA) and subcultured prior to use. Luminescent *B. anthracis* 7702-*lux* was kindly provided by Dr. T. Merkel (Food and Drug Administration, Bethesda, MD) and is described in detail elsewhere [27]. All work involving *B. anthracis* Sterne strain 7702 was performed using appropriate BSL-2 precautions. *B. anthracis* Ames strain was obtained through the NIH Biodefense and Emerging Infections Research Resources Repository, NIAID, NIH: *Bacillus anthracis*, Strain Ames (A0462), NR-411. The original stock was grown on capsulation (CAP) agar plates (0.3% yeast extract, 0.8% nutrient broth, 1.5% agar, 5% horse serum, and 0.8% sodium bicarbonate) overnight at 37°C, 5% CO_2_ in order to isolate phenotypically encapusulated organisms. Ames strain spores were prepared on agar slants as previously described [14], and bacilli were prepared fresh from CAP agar plates. All experiments with *B. anthracis* Ames strain were performed under BLS-3 precautions in a Select Agents approved laboratory following guidelines established by the Centers for Disease Control and Prevention, the US Department of Agriculture, and the University of Virginia Institutional Biosafety Committee.

### Antimicrobial assays

For CFU determination and Alamar Blue analysis, spores (0.4−1×10^6^ total) or bacilli (0.4−3×10^5^ total) were added to Dulbecco's modified essential medium (Invitrogen, Carlsbad, CA, USA) supplemented with 10% fetal bovine serum (Hyclone, Logan, UT, USA) and containing 48 µg/ml of recombinant murine or human CXCL9, CXCL10, CXCL11, CCL2, or CCL5 (Peprotech, Rocky Hill, NJ, USA) stabilized with 0.3% human serum albumin (ZLB Bioplasma AG, Berne, Switzerland), or an equal volume of albumin alone (untreated control); recombinant murine chemokines were used unless otherwise specified. Endpoint analyses were performed 6 h post-treatment (after spore germination and/or vegetative outgrowth, but before bacterial overgrowth in untreated samples) as previously described [22].

### Animal model

Wild-type C57BL/6 mice, as well as *CXCL10* ^-/-^ and *CXCR3* ^-/-^ animals were obtained from The Jackson Laboratory (Bar Harbor, ME, USA). Antibody-mediated neutralization of CXCL9, CXCL10, CXCL11, and CXCR3 was achieved using published protocols [23], [24]. Briefly, C57BL/6 mice (female, 6–8 weeks old) were administered i.p. injections of goat serum raised against recombinant CXCL9, CXCL10, or CXCL11 (R&D Systems, Minneapolis, MN, USA) or a peptide constituting the NH_2_ terminus of murine CXCR3; control animals received an equal volume of donor herd normal goat serum (SeraCare Life Sciences, Milford, MA, USA). Neutralization was begun 24h prior to spore challenge, and performed daily throughout the study period (≤20 d). For single ligand or receptor neutralization, animals received approximately 6 mg of total goat IgG daily; for multiple ligand neutralization, animals received equal amounts of the indicated neutralizing sera, approximately 15 mg of total IgG. Antibody neutralizing capacity and selectivity have been described previously [23]. Intranasal *B. anthracis* spore challenges were performed following sedation with ketamine/xylazine (60/6 mg/kg body weight, i.p.). Twenty microliters of spore suspension (1−6×10^7^ spores total) was placed drop-wise onto the nares of mice, and the animals were kept upright until breathing returned to normal. Animals were monitored for signs of illness according to an IACUC approved scoring system taking into account activity level, posture, and respiration; animals determined to be moribund were euthanized with an overdose of ketamine.

### Tissue CFU determination

All tissues used in CFU determination were harvested following euthanasia and homogenized by hand on ice in sterile PBS. Sample dilutions were prepared in duplicate, and subsequently plated on BHI agar (Remel, Lenexa, KS, USA); sample plates were incubated overnight at room temperature before colony enumeration. All tissue samples were plated ± heat treatment at 65°C for 30 min to distinguish between spore and vegetative forms of *B. anthracis*.

### Bioluminescent imaging

Images of spore-challenged mice and luminescent signals were acquired using the In Vivo Imaging System (IVIS) Spectrum (Caliper Life Sciences, Hopkinton, MA, USA). For imaging, mice were anesthetized with 2.5% isofluorane mixed with oxygen and delivered by the XGI-8 gas anesthesia system supplied with the IVIS Spectrum. Images were acquired according to the manufacturer's recommendations, and the emission of photons from live animals was analyzed using Living Image 2.5 software.

### LF detection and quantification

Functional anthrax toxin LF was measured in animal sera prepared from whole blood collected via cardiac puncture. Quantification was based on matrix-assisted laser desorption/ionization (MALDI) time-of-flight (TOF) mass spectrometry (MS) as previously described [30]. Briefly, MALDI-TOF MS was used to detect specific peptide products generated following LF-mediated cleavage of a synthetic peptide substrate; LF concentrations were subsequently determined by isotope-dilution MS.

### Statistical analysis

Significant differences among in vitro treatment groups were determined using one-way ANOVA with a Bonferroni multiple comparison post test; logarithmic (log_10_) transformation of CFU values was performed prior to statistical evaluation. The reported half maximal effective concentration (EC_50_) values were determined using the sigmoidal dose-response equation of nonlinear regression and are presented as EC_50_ ±95% confidence interval. Significant differences in bacterial counts and LF concentrations among animal treatment groups were determined using the Mann-Whitney rank-sum test for non-parametric data. Host survival was analyzed according to the Kaplan-Meier product limit method; pair-wise comparisons were made using the log-rank test.

## Supporting Information

Figure S1 CXCL9-mediated direct antimicrobial effects against *B. anthracis* Sterne strain spores and bacilli are concentration dependent. *B. anthracis* spores (A and B) or bacilli (C and D) were treated with increasing amounts of murine CXCL9 for 6 h before end point determination, n = 3 independent experiments. Alamar Blue analysis demonstrated concentration-dependent effects and was used to calculate EC_50_ values ±95% confidence interval; CFU determination supported these conclusions, (n.d.  =  none detected). For clarity, only the lowest CXCL9 concentrations demonstrating significant decreases as compared to the untreated control are labeled with asterisks; **p value <0.01, ***p value <0.001.
(0.82 MB TIF)

Figure S2 CXCR3 neutralizing serum significantly reduces host cell infiltration in response to CXCL9, CXCL10, and CXCL11 in vivo. C57BL/6 mice (n = 5 per group) received no injection (untreated) or an i.p. injection of control serum or CXCR3 neutralizing serum. Subsequently, animals received mouse serum albumin (-) or 10 ng total of each CXCL9, CXCL10, and CXCL11 (+) via i.p. injection; peritoneal lavage cytology was performed 6 h after chemokine administration. **p value <0.01, ***p value <0.001 between indicated groups.
(0.30 MB TIF)

Figure S3 CXCR3 neutralization does not disrupt CXC chemokine induction in response to *B. anthracis* spore challenge. Lung tissue (n = 5-6 animals per group per time point) was harvested from naÃ ^ve C57BL/6 mice (untreated) or spore-challenged animals receiving control serum or CXCR3 neutralizing serum. ELISA quantification is expressed as median (interquartile range) chemokine concentration measured in diluted lung homogenate filtrates.
(0.74 MB TIF)

Figure S4 Inflammatory cell populations present in the lungs of spore-challenged mice receiving CXCL9/CXCL10/CXCL11 or CXCR3 neutralizing sera are equivalent. Two days post-challenge, single cell suspensions were prepared from the lungs of C57BL/6 mice (n = 4 animals per group) receiving neutralizing or control serum. Host cell populations were analyzed by flow cytometry; the following CD45^+^ populations were examined: neutrophils (CD11b^hi^, Gr1^hi^); alveolar macrophages (CD11b^neg-lo^, CD11c^hi^), CD4^+^ T cells (CD3^+^, CD4^+^); CD8^+^ T cells (CD3^+^, CD8^+^); B cells (B220^+^, CD11c^−^); myeloid dendritic cells (CD11b^+^, CD11c^+^); airway dendritic cells (CD11c^+^, CD103^+^); inflammatory macrophages (CD11b^+^, Gr1^neg-lo^, CD11c^−^, Mac3^+^); NK cells (NK1.1^+^, CD3^−^). Results are expressed as total numbers of positive cells within the lungs.
(0.74 MB TIF)
