# Supplementary figures and images for: Interferon-Inducible CXC Chemokines Directly Contribute to Host Defense against Inhalational Anthrax in a Murine Model of Infection
Source: PLoS Pathog. 2010 Nov 18;6(11):e1001199. doi: 10.1371/journal.ppat.1001199 (PMC2987825; doi:10.1371/journal.ppat.1001199)

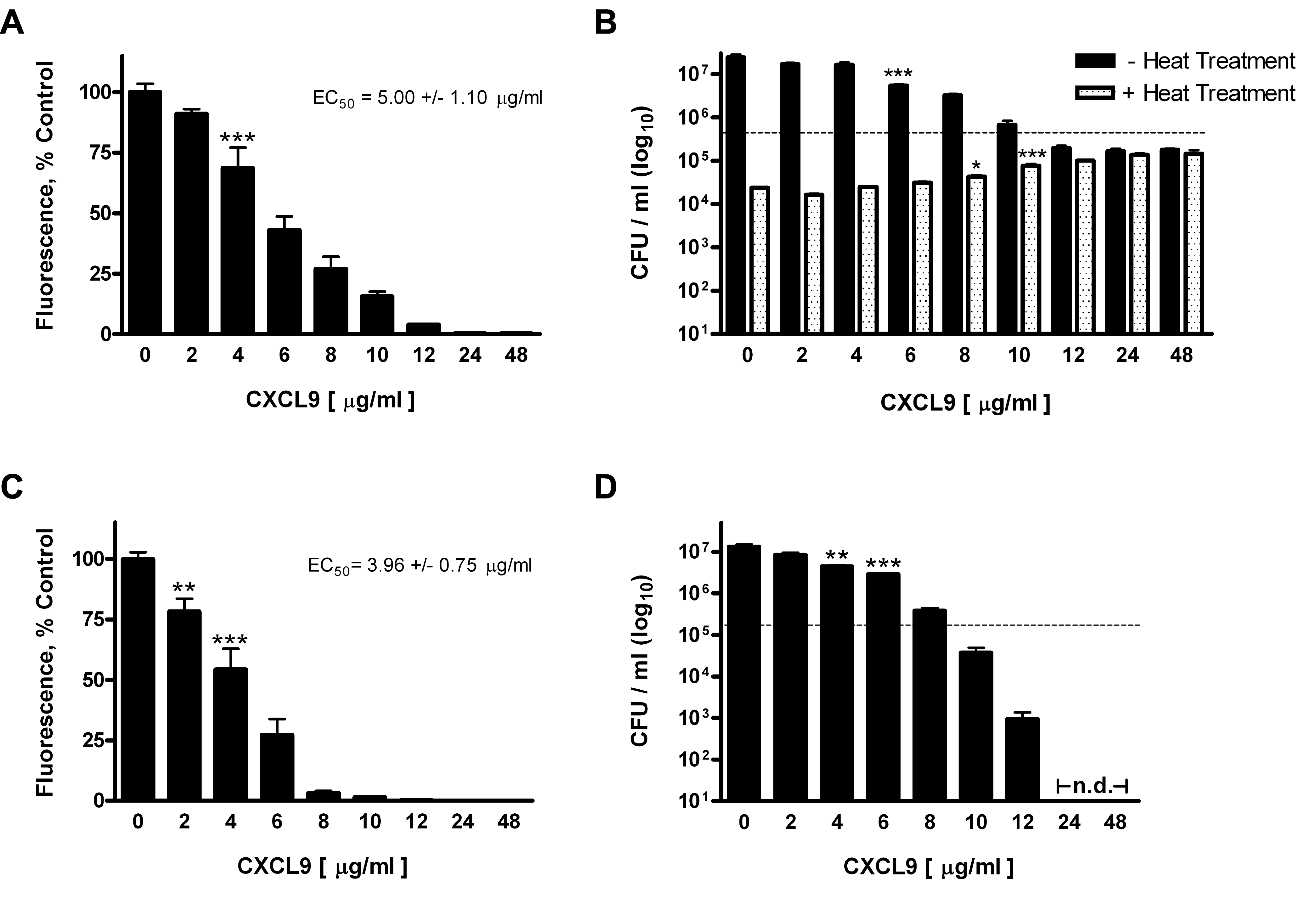

Supplement: Figure S1 — CXCL9-mediated direct antimicrobial effects against B. anthracis Sterne strain spores and bacilli are concentration dependent. B. anthracis spores (A and B) or bacilli (C and D) were treated with increasing amounts of murine CXCL9 for 6 h before end point determination, n = 3 independent experiments. Alamar Blue analysis demonstrated concentration-dependent effects and was used to calculate EC50 values ±95% confidence interval; CFU determination supported these conclusions, (n.d. = none detected). For clarity, only the lowest CXCL9 concentrations demonstrating significant decreases as compared to the untreated control are labeled with asterisks; **p value <0.01, ***p value <0.001. (0.82 MB TIF) [file ppat.1001199.s001.tif]

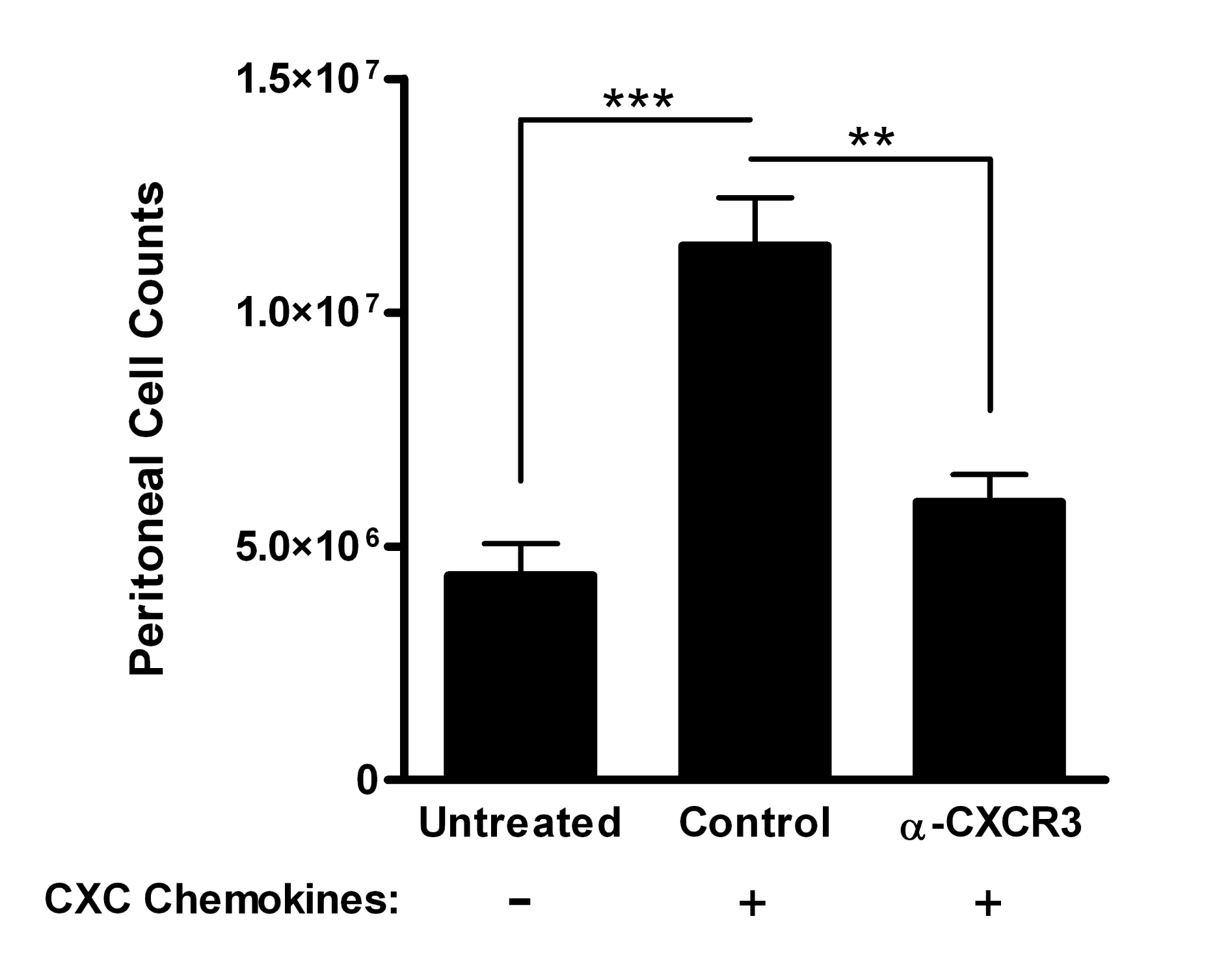

Supplement: Figure S2 — CXCR3 neutralizing serum significantly reduces host cell infiltration in response to CXCL9, CXCL10, and CXCL11 in vivo. C57BL/6 mice (n = 5 per group) received no injection (untreated) or an i.p. injection of control serum or CXCR3 neutralizing serum. Subsequently, animals received mouse serum albumin (-) or 10 ng total of each CXCL9, CXCL10, and CXCL11 (+) via i.p. injection; peritoneal lavage cytology was performed 6 h after chemokine administration. **p value <0.01, ***p value <0.001 between indicated groups. (0.30 MB TIF) [file ppat.1001199.s002.tif]

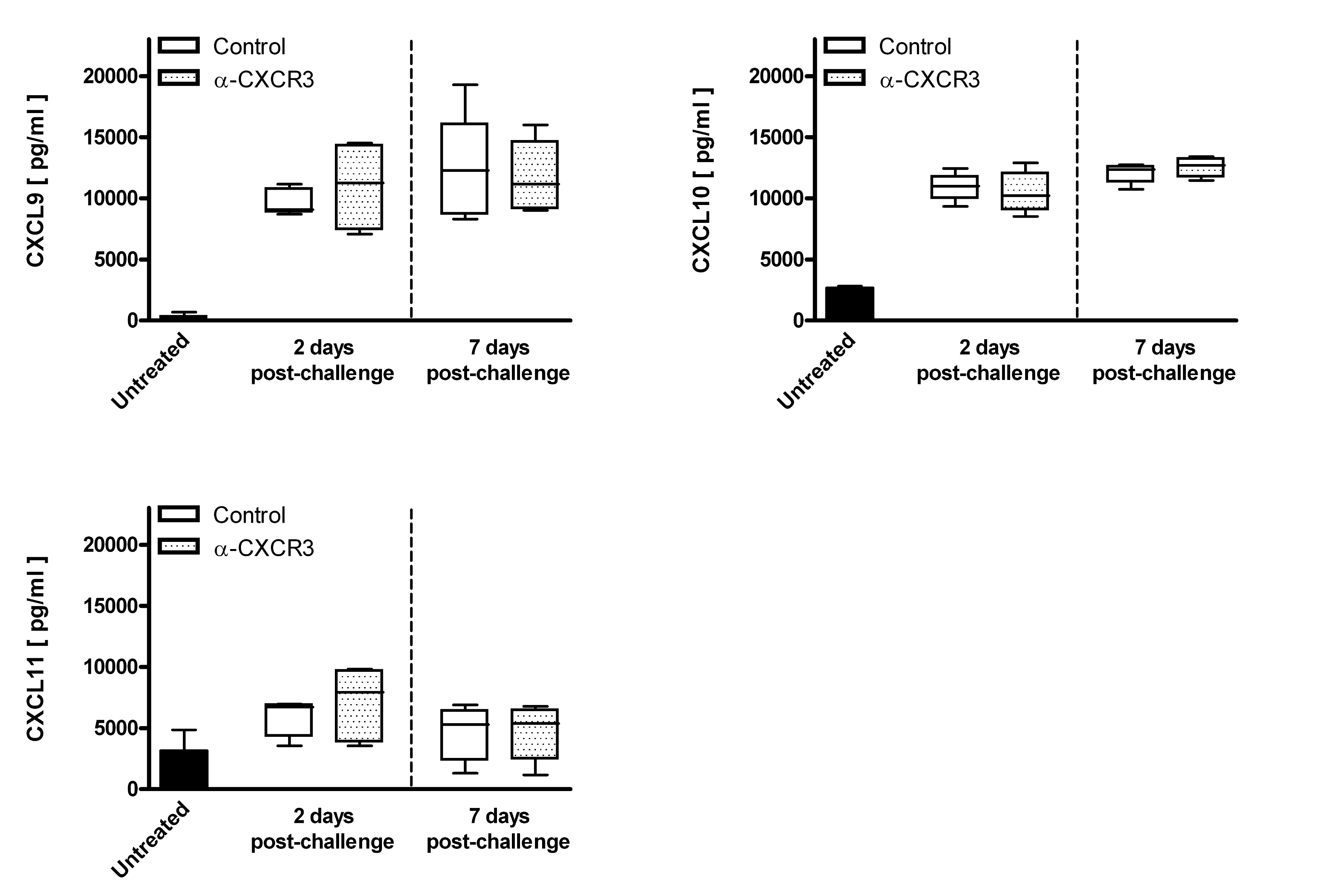

Supplement: Figure S3 — CXCR3 neutralization does not disrupt CXC chemokine induction in response to B. anthracis spore challenge. Lung tissue (n = 5-6 animals per group per time point) was harvested from naÃ ^ve C57BL/6 mice (untreated) or spore-challenged animals receiving control serum or CXCR3 neutralizing serum. ELISA quantification is expressed as median (interquartile range) chemokine concentration measured in diluted lung homogenate filtrates. (0.74 MB TIF) [file ppat.1001199.s003.tif]

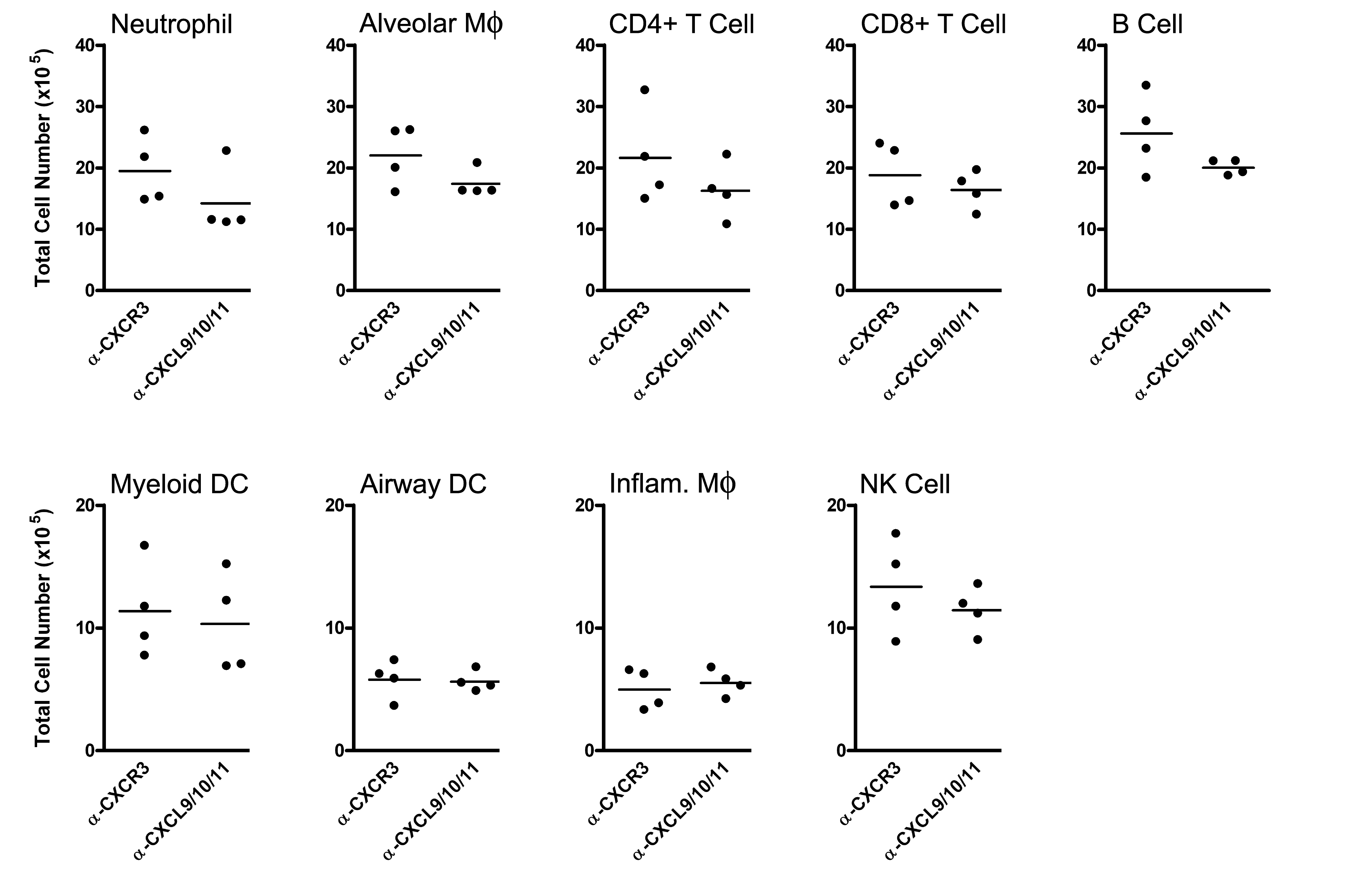

Supplement: Figure S4 — Inflammatory cell populations present in the lungs of spore-challenged mice receiving CXCL9/CXCL10/CXCL11 or CXCR3 neutralizing sera are equivalent. Two days post-challenge, single cell suspensions were prepared from the lungs of C57BL/6 mice (n = 4 animals per group) receiving neutralizing or control serum. Host cell populations were analyzed by flow cytometry; the following CD45+ populations were examined: neutrophils (CD11bhi, Gr1hi); alveolar macrophages (CD11bneg-lo, CD11chi), CD4+ T cells (CD3+, CD4+); CD8+ T cells (CD3+, CD8+); B cells (B220+, CD11c−); myeloid dendritic cells (CD11b+, CD11c+); airway dendritic cells (CD11c+, CD103+); inflammatory macrophages (CD11b+, Gr1neg-lo, CD11c−, Mac3+); NK cells (NK1.1+, CD3−). Results are expressed as total numbers of positive cells within the lungs. (0.74 MB TIF) [file ppat.1001199.s004.tif]
